# Dr. Roswell Park

**Published:** 1915-11

**Authors:** 


A tablet to the memory of the late Dr. Roswell Park has been placed in the Buffalo General Hospital, by the attending staff, commemorating his constant service from 1883 till his death in 1914. The tablet is the work of Miss Anna Glenny.

